# Institutional Transformation Reflected: Engagement in Sensemaking and Organizational Learning in Florida's Developmental Education Reform

**DOI:** 10.1007/s10755-019-09487-5

**Published:** 2019-11-04

**Authors:** Christine G. Mokher, Toby J. Park-Gaghan, Hayley Spencer, Xinye Hu, Shouping Hu

**Affiliations:** 1 Educational Leadership and Policy Studies, Florida State University, Tallahassee, FL 32306-4452, USA

**Keywords:** Developmental education, Community colleges, Institutional transformation

## Abstract

Following a major statewide developmental education reform in Florida, we explored institutional transformation among Florida College System institutions. We used statewide survey data to examine lead administrators’ perceptions of challenges encountered during the planning process, ways in which colleges engaged in sensemaking (i.e., social processes for developing shared understanding) and organizational learning, and perceptions of the institutional transformation processes and outcomes following the reform. We found that institutions engaged in numerous types of sensemaking and organizational learning practices to promote change. Yet, despite different approaches taken to institutional transformation, almost all respondents reported that the change process was highly collaborative and involved a broad range of stakeholders.

Community colleges across the nation face increasingly complex internal and external environments, resulting in an increased emphasis on the need for change (Bess and Dee 2012). One area of focus for change is developmental education, given that 68% of students attending two-year institutions must take developmental (or remedial) courses and that only 28% of these students graduate within eight years (Community College Research Center 2014). Studies have shown that developmental education may have negative impacts on students including delaying time to gateway course completion (Scott-Clayton and Rodriguez 2015) and reducing credit accumulation (Martorell and McFarlin Jr 2011). In a meta-analysis of developmental education studies using quasi-experimental methods, Valentine et al. (2017) found that students assigned to developmental education who were just below college-ready performed significantly worse on the likelihood of degree completion, credit accumulation, and passing college-level courses relative to similar students who were not assigned to developmental education. Additionally, developmental education can impact students negatively through increased costs and debt related to courses that do not provide college credit. Annually up to $7 billion is spent on developmental education expenditures nationwide by students and institutions (Scott-Clayton et al. 2014). In Florida developmental education costs were estimated at $154 million annually, with students paying $73 million through tuition (Underhill 2013).

Due to the high cost and lack of effectiveness associated with traditional developmental education programs (e.g. Scott-Clayton et al. 2014; Valentine et al. 2017), many states and college systems have responded by implementing developmental education policies intended (a) to improve assessment and placement into college courses, (b) to promote innovative instructional methods in developmental education courses that accelerate students into credit-bearing courses, or (c) to increase accountability requirements around student success (Whinnery and Pompelia 2019). For example, Minnesota, Oregon, and Washington have encouraged colleges to consider multiple measures for placement since placement tests have been shown to misplace students into developmental education courses (Ngo and Melguizo 2015; Scott-Clayton et al. 2014). California, Tennessee, and Texas have implemented new methods of instruction for developmental education such as corequisite courses where underprepared students enroll directly in introductory college-level (gateway) math and English courses and receive developmental education support at the same time. Additionally, over half of community colleges surveyed on developmental education practices indicated that they have implemented reform initiatives such as using multiple measures for course placement or shortening developmental education course sequences (Rutschow and Mayer 2018).

The reform measures in these states are largely representative of traditional state policy reform in higher education, which most commonly occurs as incremental changes over time instead of rapid and large-scale reforms (Mintrom and Norman 2013). In Florida, however, the state legislature took a more drastic approach by passing broad legislation that required all 28 Florida College System (FCS) institutions (the former community colleges) to make changes beginning in Fall 2014 to many aspects of traditional developmental education placement and instructional strategies all at one time. Prior to this legislative action, students in Florida who did not meet placement test score minimums were required to take traditional developmental education courses in mathematics, reading, or writing. For the 2013 cohort enrollment rates in developmental courses were 23% in reading, 19% in writing, and 44% in math (Hu et al. 2019). In 2013 the Florida legislature passed Senate Bill (SB) 1720: a statewide reform of developmental education with full implementation to begin in fall 2014. The first change arising from the reform was that the majority of students became exempt from placement testing and developmental education courses. Exempt students include those who entered a Florida public high school in 2003/04 or later as ninth graders and graduated with a standard high school diploma, as well as active duty military personnel. A survey of student enrollment decisions following the reform indicated that many students did elect to opt out developmental education, even if they were advised to take such courses (Park et al. 2016). However, some students who felt that they were academically underprepared continued to enroll in developmental education, particularly in math. The second change was that FCS institutions were now required to offer the remaining developmental education courses using a specific set of instructional strategies, which were to include compressed, co-requisite, contextualized, or modularized formats. Contextualized courses incorporate content in the course in an applied manner depending on the student’s major course pathway, or meta-major. The third change was that the FCS institutions were required to develop a plan to offer enhanced advising and academic support services to improve student success. Administrators reported using a variety of new practices to help students to be successful under the reform, including the use of advising tools such as early warning systems, changes to the student orientation process, and greater availability of advising resources (Woods et al. 2017). All 28 FCS institutions were affected by SB 1720, and the scale of coordination was immense. However, institutions had considerable autonomy in terms of how the changes were implemented, so there was also significant decentralized decision-making at the institutional level.

The Florida reform required significant changes to every day practice in developmental education placement and delivery. Such changes often require modifications to structural processes throughout the organization that challenge organizational ideologies and assumptions to create lasting change—a process known as *institutional transformation* (Eckel and Kezar 2003). This study contributes to the literature by providing insight into whether and how institutional transformation occurred within the FCS institutions during this large-scale developmental education reform, which required all public colleges to make several substantial changes simultaneously and quickly. We explored the changes made under the reform through the conceptual lenses of sensemaking and organizational learning. Sensemaking refers to the “social process that involves seeking information from others, collectively assigning meaning to the information, and then taking actions based on shared understandings” (Bess and Dee 2012, p. 155). This is relevant to our study since the reform required organizational members to rethink how to prepare students for success in college-level courses and then to construct a collective understanding of how they would develop innovative solutions. The concept of organizational learning refers to the process through which “the knowledge generated by individuals and groups becomes embedded within the structures, strategies, routines, and culture of the entire organization” (Bess and Dee 2012, p. 666). In our context, college administrators and staff had to create and use new knowledge about best practices in order to help students to be successful under the reform, and then share that knowledge throughout the organization.

We begin by examining the extent to which different types of resistance were encountered among different groups of stakeholders and identifying the common obstacles that arose during the initial planning process. Next, we examine the processes that FCS institutions engaged in to promote sensemaking and organizational learning during the implementation of the developmental education reform. Finally, we explore whether institutional transformation was evident in both attitudinal and structural changes and how this influenced institutional leaders’ perceptions of the reform outcomes. We conclude with implications for understanding how institutional transformation occurs following a large-scale reform, which may inform practices in other states engaged in similar initiatives.

## Theoretical Framework

We used a framework for understanding institutional change based on the work of Kezar (2018). Change is defined as “intentional acts where a particular leader drives or implements a new direction” (p. x). Sometimes change occurs in response to external conditions (e.g. enrollment declines), while other times change is a more deliberate process initiated by change agents within an organization (e.g. implementing a strategic plan). The most common obstacle to organizational change is resistance from other members of the organization. There are three main sources of resistance: (a) a lack of belief in the efficacy of the idea; (b) a lack of trustworthiness of the change agent; and (c) the existence of prior change processes that failed, resulting in cynicism. Processes that can help to reduce resistance include stakeholder participation and input, broad information sharing, full disclosure of direction and vision, trust and open communication, acknowledgement of differing values and interests, ongoing dialogue, use of transformational rather than charismatic leadership, and use of fair practices.

There are two types of changes that occur within organizations. First order (or incremental) changes are minor improvements, such as those that occur in response to fluctuations in institutional funding (e.g. Eckel et al. 2001; Keller 1983). These types of changes tend to occur more frequently and are easier to accomplish. Second order changes, which are also referred to as transformational changes, involve a more comprehensive set of changes to operational procedures, underlying values, and the culture of the organization (e.g. Gioia et al. 1996; Schein 1992). This type of change is more challenging because postsecondary institutions tend to have long-standing practices and members are not used to making significant changes. It also requires institutions to make larger collective changes instead of only changes among individuals, which is particularly difficult in higher education settings with its decentralized organizational structures and tradition of faculty governance. Since true institutional transformation occurs relatively infrequently in higher education, there are few studies that examine how this process occurs (Kezar 2018).

Social cognition theories of change are used to understand how ongoing learning occurs during the change process (Kezar 2018). This framework can inform our understanding of why transformational change is challenging and the types of barriers that may be encountered. A primary obstacle is that organizational members may not understand new initiatives or may have inaccurate assumptions about them. Two common strategies to addressing this obstacle include sensemaking and organizational learning. Weick (1995) explained sensemaking as steps that people take to search for meaning, strive and settle for plausibility, and move forward following those actions. Sensemaking is a cyclical process that begins with the question of “what’s going on here?” followed by the question of “what do I do next?” (Weick et al. 2005, p. 412). Initial steps in the sensemaking process occur through actions of “noticing” through which people communicate in order to make sense of circumstances and events that affect them and “bracketing” through which they focus on parts of the overarching problem by reflecting on the external environment. In terms of organizational change and institutional transformation, sensemaking is a process of changing people’s mindsets by providing ongoing opportunities for social interaction to introduce new ideas and promote the evolution of the thinking of organizational members (Eckel and Kezar 2003; Weick 1995). These opportunities must be widespread given the decentralized nature of decision making and implementation procedures within postsecondary institutions (Birnbaum 1988; Cohen and March 1974). Ways of creating sensemaking include having ongoing campus conversations, using collaborative leadership, developing cross-departmental teams, sponsoring development opportunities for the faculty and staff, discussing external ideas, preparing public presentations, creating documents and concept papers, and communicating a flexible vision (Kezar 2018).

The second strategy of organizational learning is an approach driven by rational thinking and data use (Kezar 2005). Organizational members create mechanisms for trying new approaches to solving a problem, learn from mistakes, and then further modify practices. Additionally, intra-organizational learning can occur when members learn from other groups that have addressed a similar problem. Organizational learning is typically acknowledged to occur through multiple levels within an organization (Dee and Leišyte 2016). It may begin with an individual sharing an insight that is then transferred to others working within the same group or team. Other members of the group may interpret and modify the information to fit the unique context of the group. In order for the learning to transfer to the organizational level, leadership is needed to identify promising group practices and develop the necessary infrastructure to implement these practices more widely. Feedback from the individual and group levels will continue to refine institutional knowledge. Ways of promoting organizational learning include introducing new ideas, distributing information, providing professional development on data use, creating groups to review and interpret data, promoting critical leadership, and valuing mistakes as learning opportunities (Kezar 2018).

There are two ways to assess whether transformational change has occurred (Kezar 2018). The first type of evidence is a change in the attitudes of organizational members, which may be seen in changes in how groups interact with each other, in the type of language used, and in individuals’ perceptions of challenges. The second type of evidence is structural change in the processes or procedures within an organization, such as the widespread use of innovative pedagogies, substantial changes in curricula, or new assessment practices. It is important to consider both types of evidence, as changes in institutional processes alone do not necessarily indicate transformational change.

As Dee and Leišyte (2016) noted, one of the most important areas for research on organizational change in higher education is understanding the mechanisms through which individual and group level changes become institutionalized at the organizational level. To further develop this literature base, we used Kezar’s (2018) framework to explore how sensemaking and organizational learning fostered institutional transformation in the context of Florida’s developmental education reform.

## The Study

### Purpose and Research Questions

This study examined institutional transformation processes in the context of the implementation of the required reform by using a system-wide survey of senior administrators’ reflections on the implementation process at different points in the past five years of policy implementation. Specifically, the study addressed the following questions:

- What challenges did colleges encounter during the initial planning process for the developmental education reform?
- What processes did colleges engage in to promote sensemaking and organizational learning during the implementation of the developmental education reform?
- How did administrators perceive the institutional transformation process and outcomes following the developmental education reform?

### Procedures

We began by analyzing survey questions regarding perceptions of various stakeholders at the beginning of the reform to identify the extent to which resistance was an obstacle to change and the processes used to help reduce resistance. Next we examined ways for creating sensemaking and organizational learning that occurred during the ongoing change process over the past 5 years at each institution following the passage of SB 1720. We concluded by assessing perceptions of institutional leaders about the extent to which attitudinal and structural changes occurred. The appendix provides a list of the survey questions and their alignment with elements from the theoretical framework.

The research plan and survey instrument had been approved by the Institutional Review Board for the Protection of Human Subjects at our University. We used FCS institutional websites to obtain email addresses for senior administrators in academic and student affairs from each of the 28 public state colleges in Florida. Each administrator received an email with a link to the online survey using Qualtrics software. The questions pertinent to this study were part of a larger survey addressing topics such as reflections on concerns at the beginning of the reform, changes over time related to the reform, and reflections on outcomes after the implementation of the reform.

Of particular interest for this study were the questions about changes in sensemaking and institutional learning practices over the past five years. This section asked administrators to identify changes to practices that happened after the passage of SB 1720 (e.g., learned about new ideas from other institutions, collected data to inform decision-making, provided opportunities for organization members to suggest new directions). Additionally, we were interested in exploring differences between expected and actual student outcomes, as well as changes over time in perceptions about supporting academically underprepared students.

### Sample

Data collection was conducted in spring 2019. Lead administrators at 21 of the 28 FCS institutions completed the survey, resulting in a response rate of 75%. We chose to focus on lead administrators for two reasons. First, lead administrators were actively engaged in a wide variety of activities under the reform, from preparing implementation plans to developing new infrastructure to reporting data on outcomes. This broad level of participation allowed them to see different practices used in the change process throughout the organization. Second, leadership is needed to ensure that changes at the individual or group level become institutionalized, so these lead administrators were in the best position to assess whether changes had occurred throughout the organization. Even though the respondents provide a single perspective for each institution, some organizational learning scholars have noted that the significant influence that top-level managers have on organizational strategy “can serve as a proxy for understanding the ways in which an organization ‘thinks’” (Dee and Leišyte 2016, pp. 286–287).

The instructions for the survey indicated that only one response was needed for each institution. Those who received the email were advised that they could select a designee to complete the survey or work collaboratively with other leaders at their institution. The majority of the respondents were vice presidents or assistant/associate vice presidents (66%), and the remaining responses were completed by provosts or other administrators such as deans and academic officers (34%). Several respondents did not answer every question, so the figure notes in the findings section indicate the number of responses for each of the specific questions.

### Data Analysis

We conducted a comprehensive descriptive analysis of the survey data. We categorized the results into three areas that correspond with the research questions: 1) initial obstacles to the planning process, 2) sensemaking and organizational learning processes during implementation, and 3) reflections on institutional transformation and outcomes. Most of the questions were on a five-point scale such as “not at all” (1) to “to a great extent” (5). For some questions respondents were asked about how perceptions differed among different groups of stakeholders including lead administrators, faculty, academic support staff, student support services staff. Even though respondents may have been able to report most accurately from the perspective of lead administrators, they are likely to have had a sense of how perceptions may have differed among other stakeholders due to their frequent interactions with other groups throughout the implementation process. The responses to these questions were tabulated across institutions and the results were presented in figures. Additionally, the survey included open-ended questions about changes leaders would make if they had to start over again with the developmental education reform, examples of ways in which perceptions changed following the reform, and general feedback about reform implementation. In the findings section, we include quotes from the open-ended survey questions so as to provide additional evidence in support of some of the quantitative findings.

## Findings

We present the findings in three sections, the first of which provides reflections on the initial planning process. Next, we explain the findings that relate to sensemaking and organizational learning processes during implementation of SB 1720. We then explore the reflections on institutional transformation and outcomes.

### Challenges Encountered during the Initial Planning Process

**Fig. 1:**
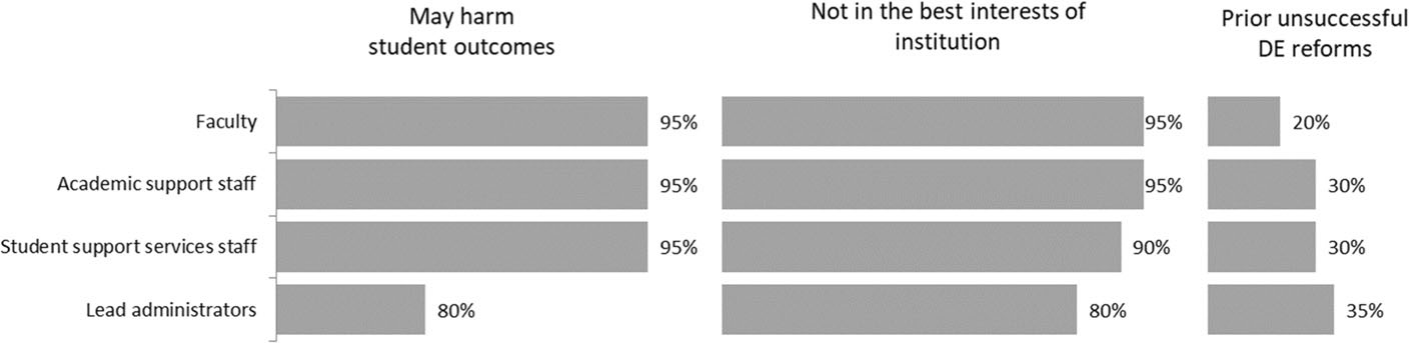
Percentage of respondents indicating that stakeholder groups expressed initial concerns to a “moderate” or “great” extent as perceived by the respondents. Scale ranges from 1 (not at all) to 5 (to a great extent). N = 20 institutions

Since prior literature has identified resistance from organizational members as one of the most common obstacles to change (e.g. Kezar 2018), we began by exploring potential sources of resistance due to a lack of belief in the efficacy of the idea, a lack of trustworthiness of the change agent, and cynicism from prior change processes that failed. Figure 1 displays the extent of initial concerns about SB 1720 among four stakeholder groups: the faculty, academic support staff, student support services personnel, and lead administrators, as perceived by the respondents to the survey. Ninety-five percent of respondents indicated that the faculty, academic support staff, and student support services personnel were concerned that SB 1720 would harm student outcomes to a moderate or great extent. Similarly, 95% of respondents indicated that faculty and academic support staff were concerned that developmental education reform was not in the best interests of the institution to a moderate or great extent. Related to the best interests of students and the institution, one respondent stated, “There was (and remains) a great deal of skepticism that the change was really in the students’ best interests.” Another administrator echoed this concern, saying:

I would say that, as thorough as our institutional planning process was and as many stakeholders … were involved as possible, there was (and remains) a great deal of skepticism that the change was really in the students' best interests. Perhaps we could have/should have done more with getting [a] larger number of our own staff to participate in the statewide conversations during that initial planning process, rather than relying on a few key emissaries.

Lead administrators had fewer concerns regarding student outcomes and the best interests of the institution than did the other stakeholders; however, they had the highest level of concern arising from prior unsuccessful developmental education reform attempts.

**Fig. 2:**
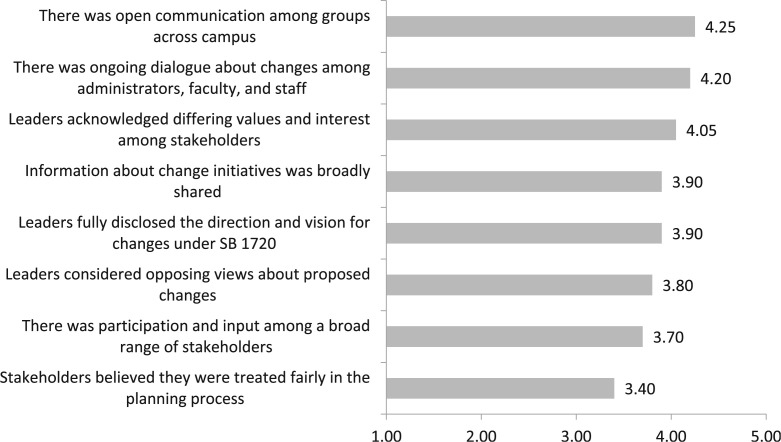
Mean score for agreement with statements about collaboration in the initial planning process for SB 1720. Scale ranges from 1 (strongly disagree) to 5 (strongly agree). N =20 institutions

Figure 2 displays the agreement with statements regarding the extent of collaboration during the initial planning process on a scale from 1 (strongly disagree) to 5 (strongly agree). Overall the mean scores show that there was little disagreement with the statements below, with means ranging from 3.40 to 4.25, indicating that respondents perceived the change process to be very collaborative. The levels of highest agreement were with statements related to communication among groups across campus and ongoing dialogue among administrators, faculty members, and staff (mean of 4.25 and 4.20, respectively). The statements with the lowest agreement mean that scores were related to participation and input among a broad range of stakeholders (mean= 3.70), and that stakeholders believed they were treated fairly in the planning process (mean = 3.40). As one administrator noted:

Over the past six years we have worked collaboratively with faculty and other stakeholders in implementing the requirements of Senate Bill 1720. Our Developmental Education Council… has been an especially helpful voice for discussing and improving policies and procedures related to developmental education curriculum, instruction, assessment, as well as academic and student support services.

### Processes to Promote Sensemaking and Organizational Learning

**Fig. 3:**
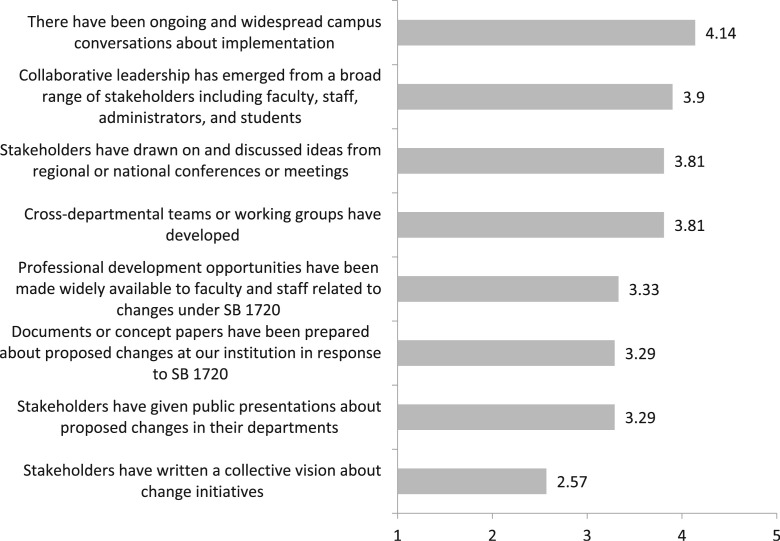
Mean score for reflections on sensemaking during the ongoing change process over the past 5 years at your institution following SB 1720. Scale from “not at all” (1) to “to a great extent” (5). *N* = 21 institutions

We found that the institutions engaged in a variety of different sensemaking activities, as shown in Fig. 3. Communication and collaboration continue to be a large part of the change process with “moderate” occurrences of ongoing and widespread conversations about implementation (mean = 4.14) and a “moderate” extent of collaborative leadership emerging from faculty, staff, administrators, and students (mean =3.9). Sharing information also occurred in professional development activities and public presentations, which happened occasionally (mean = 3.33 and mean = 3.29, respectively). The creation of a written collective vision about change initiatives appears to be have happened to a lesser extent than did other aspects of the ongoing change process (mean = 2.57). As one respondent stated:

Looking back, we exceeded our own expectations in planning for and implementing SB1720. Doing [it] all over again, we may have done more to carefully document our work and publish our successes and challenges in this initiative for wider public consumption.

Seventeen respondents offered open responses and noted different areas of collaboration and communication with stakeholders; however, four of these respondents also noted that time constraints during implementation made this process more difficult. One respondent stated: “[The] timeline for implementation was unrealistic and created a lot of stress during implementation.” Others were more optimistic as in this comment: “We actually did a good job, given the aggressive timeline from the state, including campus stakeholders once SB 1720 became law.

**Fig. 4:**
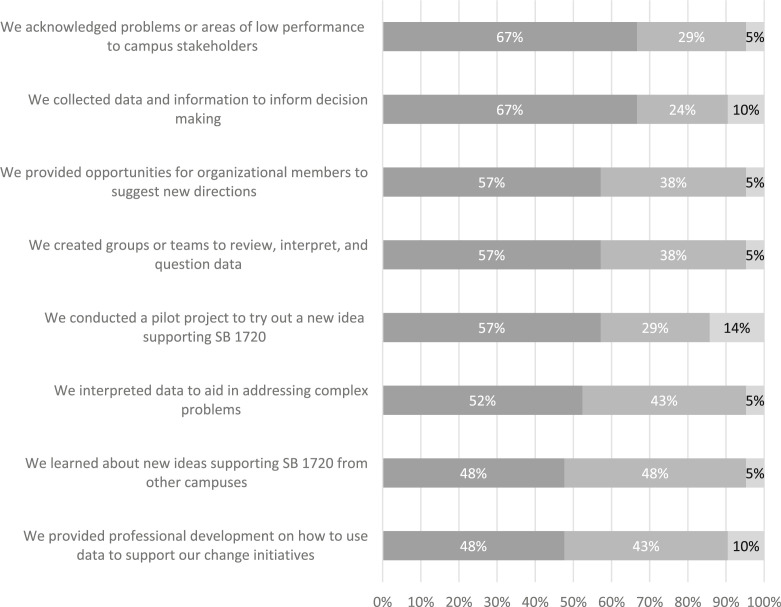
Percentage of respondents indicating that their institution engaged in organizational learning practices occurring over the last 5 years after SB 1720 implementation. Scale ranges from “this usually happened on my campus” to “this sometimes happened on my campus” to “this did not happen on my campus.” *N* = 21 institutions

Figure 4 displays the percentage of respondents indicating that different organizational learning practices associated with the ongoing change process occurred at their institutions over the past five years following implementation of SB 1720. All eight of the organizational learning practices occurred at most institutions at least “sometimes” over the past five years. The practices that occurred most often were acknowledging problems or areas of low performance and collecting data and information to inform decision making (67% for both).

The practice that occurred least frequently was conducting a pilot project to try out a new idea supporting SB 1720 (14%). Five to 10 % of the institutions reported that “other organizational learning practices” never occurred. Some administrators later came to regret not engaging in these practices, and one respondent commented, “We adopted compressed classes only. I wish we had experimented with co-req[uisite] or contextualized options.”

### Perceptions of the Institutional Transformation Process and Outcomes

As we discussed earlier, transformational change requires both structural and attitudinal changes. To assess structural change, respondents were asked to rate the extent to which changes had been made in six areas of student services practices, developmental education courses, and gateway courses over the past five years. Respondents reported that the greatest areas of change were in advising, developmental education instructional practices, developmental education curricula, and student support services. The extent of change in these types of practices is not surprising given that the legislation mandated changes in these areas. Yet there were also at least “moderate” changes to instructional practices and curricula in gateway courses even though these types of changes were not required by the legislation. In each of these areas of change, over 80% of respondents indicated that these changes were at least “somewhat” attributed to SB 1720. The open-ended responses provided further information on the types of changes that had been made. For example, one respondent stated that the institution “has implemented numerous changes from SB1720, including course revisions, staffing adjustments, enhancements to academic support centers and student affairs, scheduling changes, and implementation of early alert systems.”

**Fig. 5:**
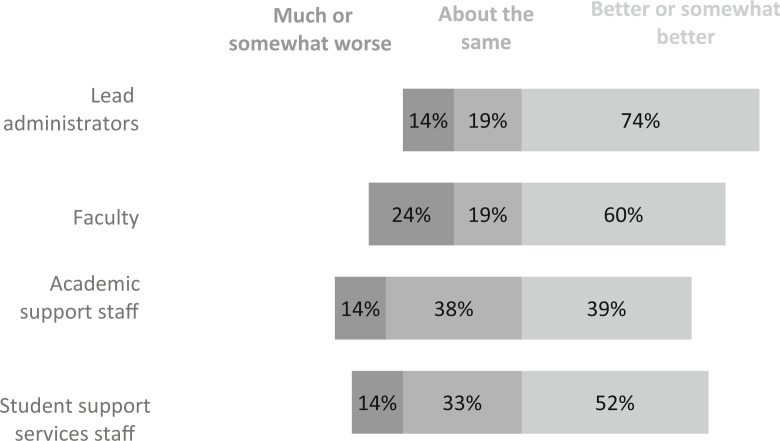
Mean scores for perceptions of performance on student outcomes compare to initial expectations at the beginning of SB 1720 as perceived by the survey respondents. Scale from “much worse than anticipated” (1) to “much better than anticipated” (5). N = 21 institutions

Respondents were also asked to rate how they believed the perceptions of performance on actual student outcomes compared to initial expectations at the beginning of SB 1720 among the groups of lead administrators, faculty, academic support staff, and student support services personnel. The percentage of respondents who indicated that actual outcomes were “better” or “somewhat better” than anticipated ranged from 48% for academic staff to 67% for lead administrators (Fig. 5). As one respondent noted:

This was a tremendous example of how large scale change can be made in the state and within an institution. Ultimately, the changes to our curriculum have been beneficial. Large scale change can take effect in a year or so. It needs to be monitored closely using data and the voices of stakeholders. It is an interesting example of initiating large, scale, statewide change within very specific guidelines given to the state colleges. This holds out hope that large scale change can [indeed] occur and [can happen] pretty quickly if the parameters are clear.

However, 14% to 24% of respondents estimated that stakeholders perceived that performance on student outcomes was “somewhat” or “much” worse than anticipated. One respondent explained, “We see a persistent gap in course completion rates in gateway courses between exempt students and non-exempt students; exempt students continue to struggle in their first year in gateway courses and require extra outreach and support.” Administrators perceived that faculty members are most likely to report “somewhat worse” or “much worse” performance on student outcomes than expected. When respondents were asked if they would change anything about their planning or implementation process, one responded, “At all levels, the college as a whole should have taken more proactive steps that would help (not hurt) the students affected by the bill.”

**Fig. 6:**
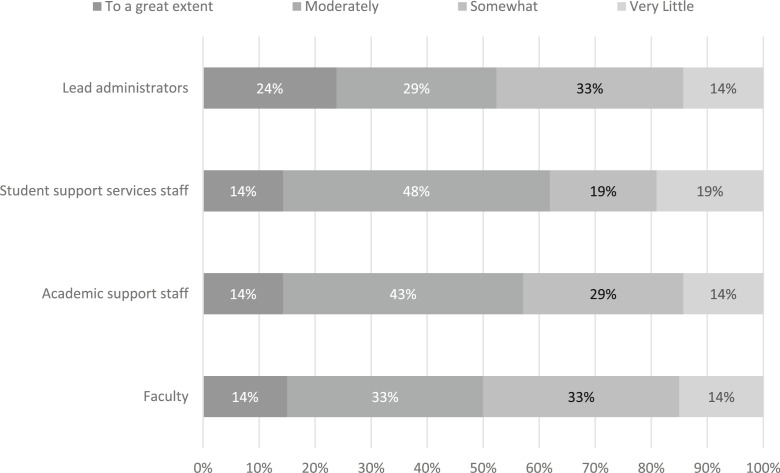
Percentage of respondents indicating changed perceptions following SB 1720 among different groups of stakeholders about the most effective ways to help students who are underprepared academically as perceived by the respondents of the survey. Responses ranged from “not at all” (1) to “to a great extent” (5). N = 21 institutions

In order to assess whether attitudinal changes occurred, respondents’ were asked to report about the extent to which perceptions changed following SB 1720 relating to the most effective ways to help academically underprepared students. We disaggregated the results so as to reflect changes among student support services staff, academic support staff, faculty members, and lead administrators. As shown in Fig. 6, at least some change in perception occurred for every group, but there were some differences in the extent of change. Among all four groups the majority of responses indicate that perceptions changed at least “somewhat”, with fewer than 20% of respondents indicating that there was “very little change.” Lead administrators noted the largest degree of change, with 24% of respondents indicating that they changed their perceptions “to a great extent.” In the open-ended questions, respondents indicated that some of the ways in which the perceptions of lead administrators changed include switching from a focus on extensive remediation to just-in-time intervention, increasing alignment with best practices, using a trial and error approach to provide support systems to understand the strategies needed to address students’ individual needs, and realizing that testing may not have been as connected to student ability as initially perceived.

Despite the majority of positive responses, 14% to 19% of respondents noted that stakeholder groups experienced little change in perceptions for supporting academically underprepared students. The open-ended responses indicated that this lack of change may be due to insufficient time or resources to implement all of the desired changes. One respondent indicated that instructors perceived little change because, “For math, there is still a feeling that it is a race against time to get the content mastered in the current forms.” Another respondent indicated that “the [student support services] staff understand that students need even more support now, but there are challenges with lack of resources.

## Discussion, Implications and Future Research

The results from our survey of lead administrators revealed several insights about the institutional transformation process during Florida’s developmental education reform. First, we found that, while FCS institutions faced a variety of challenges during the initial planning process, collaboration among stakeholders was an area of strength. Survey respondents reported that prior to SB 1720, four stakeholder groups had expressed concerns that the reform may harm student outcomes, may not be designed in the best interests of their institutions, or may face a fate similar to prior unsuccessful reform attempts. Yet, despite these concerns, the overall finding showed that stakeholders came together to work collaboratively toward institutional change. Most respondents reported open communication among groups across campus and ongoing dialogue about changes. We suggest that this level of collaboration is somewhat surprising given that postsecondary institutions have historically struggled to engage in organizational-wide learning due to extensive decentralization, structural differences across department and administrative units, and cultural differences across disciplines and roles (Dee and Leišyte 2016).

Second, we found that institutions engaged in sensemaking and organizational learning processes in a variety of different ways during implementation of the reform. Most institutions used a variety of different approaches to sensemaking; the most common ones were having ongoing campus conversations about implementation, engaging in collaborative leadership among the stakeholder groups, and discussing ideas from regional or national conferences or meetings. Similarly, most institutions tried various organizational learning practices, the most prevalent of which were acknowledgement of areas of low performance and collection of data to inform decision making. The use of multiple organizational learning practices is consistent with prior research, which has shown that there must be intentional opportunities for individuals throughout the organization to engage in activities that lead to new ideas and thinking in order for transformational change to occur (e.g. Eckel and Kezar 2003).

Third, the reform was largely perceived to have changed attitudes of stakeholders throughout the institution and resulted in changes that improved student success. For the most part, respondents indicated that stakeholder groups saw “better” or “somewhat better” performance on student outcomes compared to initial expectations and that there were at least “moderate” changes in perceptions about the most effective ways to help underprepared students. These positive perceptions are aligned with results from our quantitative evaluation of the reform, which indicate that there has been a statewide increase in student success as measured by the passing rates for introductory college-level courses based on student cohort and college-level credit hours attempted and earned during the first year of enrollment (Hu et al. 2019). Lead administrators were the most optimistic about the outcomes of SB 1720, with two-thirds of respondents indicating that this groups saw “better or somewhat” better performance than expected. Yet there were some who perceived that performance was “somewhat” or “much worse” than anticipated; respondents reported that this worsening perception was most common among faculty members. Similarly, respondents reported that changes in perceptions about the most effective ways to help academically underprepared students were greatest among lead administrators and least among faculty members.

Taken together, these findings suggest that FCS institutions have engaged in institutional transformation by making comprehensive changes to operational procedures, as well as demonstrating changes to underlying values and beliefs among broad groups of stakeholders. The presence of both structural and attitudinal evidence is important for ensuring that institutions move beyond first order changes to higher-level second order, or transformational, changes. In line with our social cognition theory of change, we found that institutions engaged in different types of sensemaking and organizational learning practices to promote change. We also found that almost all respondents reported that the change process was highly collaborative. The collaborative nature of activities is important for ensuring that change becomes institutionalized throughout the organization rather than remaining isolated within departments or among individuals (Kezar 2018).

We believe that this study has implications for both practice and research. In practice, leaders should consider how they can initiate or encourage sensemaking and organizational learning activities to help facilitate change. For example, the results from this study indicated that most administrators perceived that there was widespread conversation across campus about implementation to a “moderate” extent (mean of 4.14 on a scale of 5.0). The greater challenge was converting conversations to action. Fewer respondents reported actions like developing concept papers, giving public presentations, or writing a collective vision (mean of 3.33 or less on a scale of 5.0). Leaders may want to promote these types of activities early in the implementation process so as help to overcome skepticism that different stakeholder groups may express.

We are continuing our evaluation of Florida’s developmental education reform, which includes extensive qualitative and quantitative analyses to further understand the change process and the extent to which these changes may have resulted in improvements in student outcomes. For example, organizational learning is driven by rational thinking and data use, but little is known about data cultures within institutions of higher education. One of our qualitative studies is using data from interviews with institutional leaders in order to understand how administrators share data with frontline staff in community colleges and how those data are used to identify and solve organizational problems that may impede efforts at institutional improvement. Additionally, our future work will seek to link institutional differences in data cultures and practices quantitatively with student outcomes in Florida’s state colleges. We anticipate that institutions with higher levels of data sharing may expect to see higher performance due to increased operational efficiency, improved work environments, greater flexibility, and collaborative problem solving. These types of studies will further contribute to understanding how organizational change occurs and its implications for improving student success.

## Conclusion

Institutions in Florida faced a sizeable task when the legislature passed SB1720; however, this task seems to have been met with a sense of collaboration and community as the institutions transformed to meet the requirements of the legislation and develop practices intended to promote student success. Florida provides an example of how legislative reform of developmental education, even if met with initial concern or skepticism, can be effective at promoting positive change and can contribute to student success.

## Appendix

**Table:** Alignment of Theoretical Framework Elements and Survey Questions.

Theoretical framework element	Survey question
Most common obstacles to change due to resistance	To what extent did the following groups express concerns that SB 1720 may harm student outcomes? 1) Not at all, 2) Very little, 3) Somewhat, 4) Moderately, 5) To a great extent:
	a) Lead administrators, b) Faculty, c) Academic support staff, d) Student support services staff.
	To what extent did the following groups express concerns that state policymakers designed SB 1720 in a way that was not in the best interests of your institution? 1) Not at all, 2) Very little, 3) Somewhat, 4) Moderately, 5) To a great extent:
	a) Lead administrators, b) Faculty, c) Academic support staff, d) Student support services staff.
	To what extent did the following groups express concerns that there had been prior unsuccessful attempts at reforming developing education at your institution, leading to skepticism about SB 1720? 1) Not at all, 2) Very little, 3) Somewhat, 4) Moderately, 5) To a great extent:
	a) Lead administrators, b) Faculty, c) Academic support staff, d) Student support services staff.
Processes to help reduce resistance	To what extent do you agree with the following statements about the initial planning process for SB 1720? 1) Strongly disagree, 2) disagree, 3) neutral, 4) agree, 5) strongly agree: There was participation and input among a broad range of stakeholders. Information about change initiatives was broadly shared. Leaders fully disclosed the direction and vision for changes under SB 1720. There was open communication among groups across campus. Leaders acknowledged differing values and interest among stakeholders. There was ongoing dialogue about changes among administrators,faculty, and staff. Leaders considered opposing views about proposed changes. Stakeholders believed they were treated fairly in the planning process.
Ways of creating sensemaking	To what extent do the following statements reflect the ongoing change process over the past 5 years at your institution following SB 1720? 1) Not at all, 2) Very little, 3) Somewhat, 4) Moderately, 5) To a great extent: There have been ongoing and widespread campus conversations about implementation. Collaborative leadership has emerged from a broad range of stakeholders including faculty, staff, administrators, and students. Cross-departmental teams or working groups have developed. Professional development opportunities have been made widely available to faculty and staff related to changes under SB 1720. Stakeholders have drawn on and discussed ideas from regional or national conferences or meetings. Stakeholders have given public presentations about proposed changes in their departments. Documents or concept papers have been prepared about proposed changes at our institution in response to SB 1720. Stakeholders have written a collective vision about change initiatives.
Ways of creating organizational learning	Which of the following practices occurred over the past 5 years at your institution following SB 1720? 1) This usually happened on my campus, 2) this sometimes happened on my campus, 3) this did not happen on my campus: We learned about new ideas supporting SB 1720 from other campuses. We conducted a pilot project to try out a new idea supporting SB 1720. We collected data and information to inform decision making. We provided professional development on how to use data to support our change initiatives. We interpreted data to aid in addressing complex problems. We created groups or teams to review, interpret, and question data. We provided opportunities for organizational members to suggest new directions. We acknowledged problems or areas of low performance to campus stakeholders.
Structural evidence of institutional transformation	To what extent have changes been made in the following areas over the past five years? 1) Not at all, 2) very little, 3) somewhat, 4) moderately, 5) to a great extent: Instructional practices in developmental courses Instructional practices in gateway courses Curriculum in developmental courses Curriculum in gateway courses Advising practices Student support services For each of the following groups, how did actual performance on student outcomes compare to initial expectations about what might happen to student outcomes at the beginning of SB 1720? 1) Much worse than anticipated, 2) somewhat worse than anticipated, 3) about the same, 4) somewhat better than anticipated, 5) much better than anticipated: Lead administrators Faculty Academic support staff Student support services staff
Attitudinal evidence of institutionaltransformation	To what extent have perceptions changed following SB 1720 about the most effective ways to help students who are underprepared academically among the following groups? 1) Not at all, 2) very little, 3) somewhat, 4) moderately, 5) to a great extent: Lead administrators Faculty Academic support staff Student support services staff
